# Molecular Diagnosis and Prenatal Phenotype Analysis of Eight Fetuses With Ciliopathies

**DOI:** 10.3389/fgene.2021.705808

**Published:** 2021-10-05

**Authors:** Yuefang Liu, Hui Wang, Xin Jin, Qixiang Shao, Qiong Pan

**Affiliations:** ^1^ Department of Clinical Genetics, Huai’an Maternity and Child Clinical College of Xuzhou Medical University, Huai’an, China; ^2^ Reproductive Sciences Institute, Jiangsu University, Zhenjiang, China; ^3^ Department of Immunology, Jiangsu Key Laboratory of Medical Science and Laboratory Medicine, School of Medicine, Zhenjiang, China; ^4^ Jiangsu College of Nursing, School of Medical Science and Laboratory Medicine, Huai’an, China

**Keywords:** ciliopathies, prenatal clinical phenotype, occipital encephalocele, polydactyly, polycystic kidneys, whole-exome sequencing

## Abstract

Human ciliopathies are hereditary conditions caused by variants in ciliary-associated genes. Ciliopathies are often characterized by multiple system defects. However, it is not easy to make a definite diagnosis in the prenatal period only based on the imageology. In this report, eight new prenatal cases from five unrelated families diagnosed with ciliopathies were systematically examined. The clinical manifestations of these fetuses showed such prenatal diagnostic features as occipital encephalocele, and polydactyly and polycystic kidneys. *Situs inversus* caused by *CPLANE1* variant was first reported. In Family 1 and Family 3, homozygous variants of *CPLANE1* and *NPHP4* caused by consanguineous marriage and uniparental disomy were detected by whole-exome sequencing, respectively. In Family 2, Family 4 and Family 5, compound heterozygotes of *TMEM67* and *DYNC2H1* including two novel missense variants and one novel nonsense variant were identified. The distribution of pathogenic missense variants along *TMEM67* gene mainly clustered in the extracellular cysteine rich region, extracellular area with unknown structure, and the transmembrane regions. Genotype-phenotype relationship between C*PLANE1* and *TMEM67* genes was concluded. This report describes new clinical manifestations and novel variants in *CPLANE1*, *TMEM67*, *NPHP4*, and *DYNC2H1*.

## Introduction

Primary cilia are highly conserved organelles located on the surface of almost all polar cells, which play important roles in tissue morphogenesis, and chemical and mechanical signal transduction (Hirokawa et al., 2006; Copeland, 2020). Genetic variants affecting the structure or function of primary cilia can lead to a broad range of developmental diseases known as ciliopathies. The whole-exome sequencing (WES) has been widely used in the clinical molecular diagnosis of ciliopathies in adults and children. Presently, approximately 187 established ciliopathy-related genes have been identified in humans, variants in which can be associated with 35 ciliopathy syndromes (Reiter and Leroux, 2017; McConnachie et al., 2021). It has been clarified that variant in *CPLANE1* causes Joubert syndrome (JBS; MIM#614615), which is characterized by a unique cerebellar and brainstem malformation, also known as molar tooth sign (MTS). Moreover, *TMEM67*-related ciliopathies are mainly JBS and Meckel syndrome (MKS; MIM#607361). While MKS is a lethal disorder with typical renal cystic dysplasia, polydactyly, and occipital encephalocele. Variants in *NPHP4* cause nephronophthisis (NPH; MIM#606966) characterized by end-stage renal disease in the first 2 decades of life. In addition, the typical feature of short-rib thoracic dysplasia (SRTD; MIM#613091) caused by *DYNC2H1* variants is skeletal dysplasia. However, the different etiology of ciliopathies and the wide range of genetic variations lead to phenotypic variability. It is challenging to choose appropriate molecular testing in the prenatal period. Therefore, it is important to have a comprehensive understanding of the prenatal phenotypes of different ciliopathy syndromes before the WES testing.

Here we report the typical and atypical features of eight fetuses with pathogenic variants in *CPLANE1, TMEM67, NPHP4*, and *DYNC2H1* found *via* WES. New clinical manifestations and the discovery of novel genetic variants are helpful for the prenatal diagnosis of ciliopathies.

## Patients and Methods

### Patients

Eight fetuses from five unrelated families diagnosed with ciliopathies were collected. Informed consents for research investigations were obtained from the relatives of the fetuses. The research protocol was approved by the local ethics committee of Jiangsu Huai’an Maternity and Child Healthcare Hospital (2019036).

### Whole-Exome Sequencing

The whole exomes were captured by using Agilent’s SureSelect Whole Exome Gene Detection Kit. High-throughput sequencing was performed by using the Novoseq sequencer from Illumina. The obtained sequence was aligned on the human genome GRCh37/hg19 reference sequence by BWA (Burrows-Wheeler Aligner) software. A BAM (binary sequence alignment map format) file was produced *via* Picard software. GATK4 (Genome Analysis Toolkit) Realigner Target Creator software and Haplotype Caller software were used to adjust the sequence, extract variants, and generate VCF (Variant Call Format) files. The Annovar software was used to filter and annotate the variant.

### Analysis of Variants

All nonsense, frameshift, and canonical splice site variants were considered to be deleterious. The pathogenic potential of missense variants was predicted by PolyPhen2, SIFT, PROVEAN, and Mutation Taster. The frequency of putative variants was obtained from the Human Gene Mutation Database (HGMD), Genome Aggregation Database (gnomAD), and the 1000 Genomes (1000G) database. Conservation of mutated amino acid residues in different species was compared by UCSC. SpliceAI was used to evaluate a destroyed splice site. The deleteriousness of variants was assessed according to American College of Medical Genetics (ACMG) standards and guidelines.

### Sanger Sequencing

Sanger sequencing was performed to confirm suspected variant segregation within probands’ family, and the authenticity of variants identified by WES. Primer 5 was used for primer designs. Target DNA of the fetus and its parents was amplified by PCR. Sanger sequencing results were compared with standard sequence in GenBank by SeqMan software.

## Case Presentation

### Family 1

**FIGURE 1:**
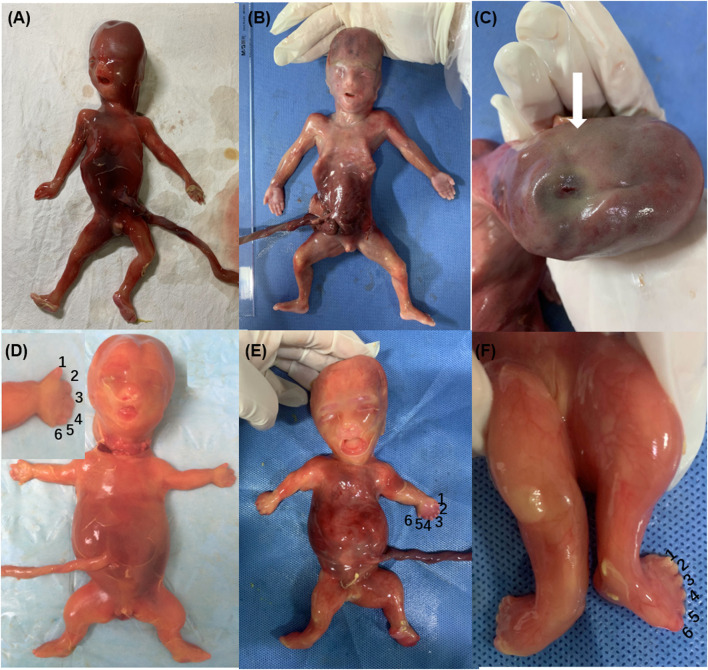
Phenotypes of ciliary diseases {Family 1 [**(A)**: case 1; **(B,C)**: case 2], Family 4 [**(D)**: case 6], Family 5 [**(E,F)**: case 8], occipital encephalocele **(C)**, polydactyly of hands and feet **(D–F)**, short limb and ribs **(D,E)**}.

**FIGURE 2:**
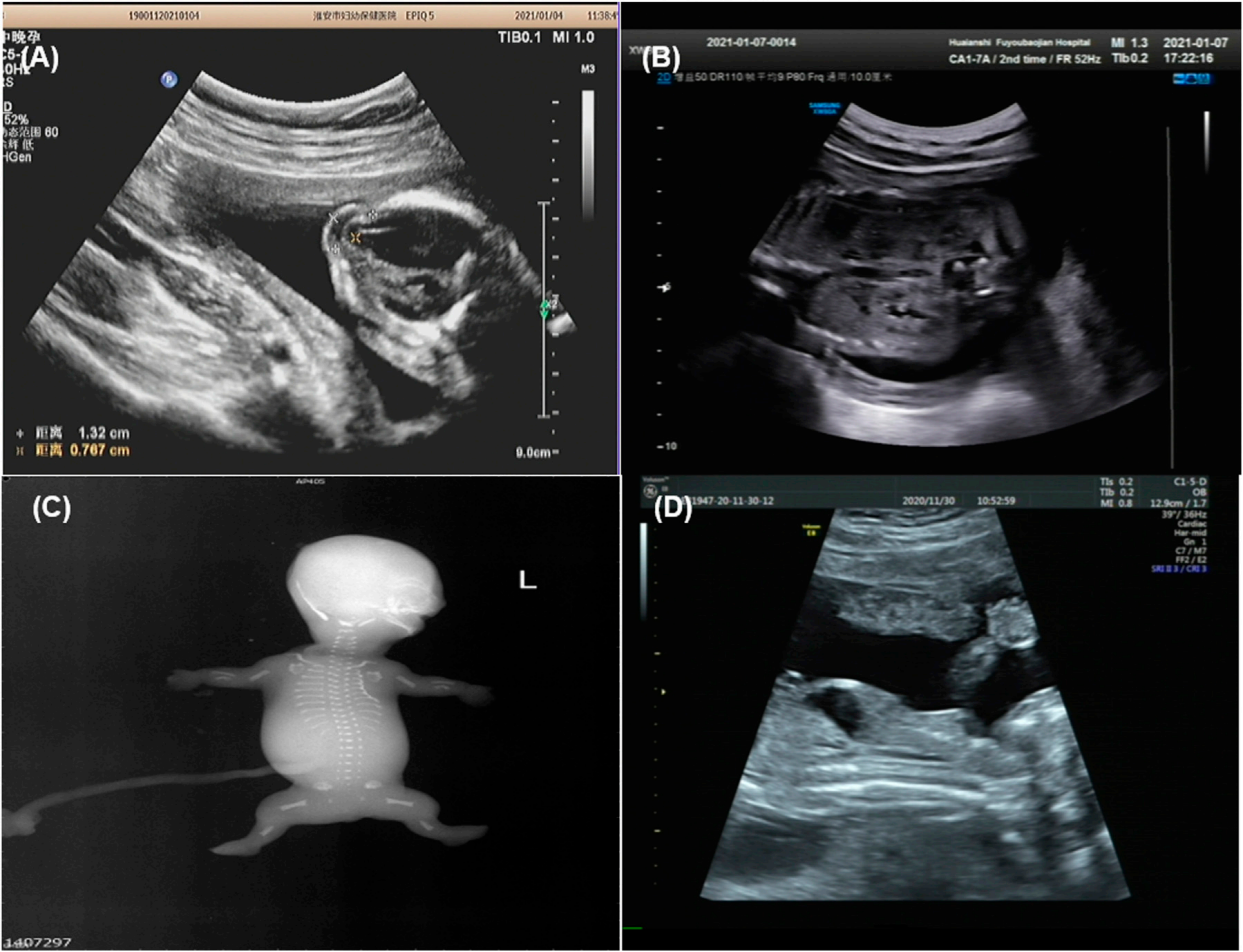
Imaging examination of ciliary diseases {Family 1 [**(A)**: case 2], Family 3 [**(B)**: case 5], Family 4 [**(C)**: case 6], Family 5 [**(**D**)**: case 8], occipital encephalocele **(A)**, increased echogenicity in both renal cortexes **(B)**, fetal X-ray showing bilateral shortened curved femora and disproportionately shortened tibiae **(C)**, bone dysplasia and narrow chest **(D)**}.

Family 1 was a consanguineous marriage with a healthy girl and five adverse obstetric outcomes. In addition to two early miscarriages, the third time was due to a widened posterior fossa and vermis hypoplasia at the 27th week of pregnancy without genetic detection. Case 1 was the fifth pregnancy, whose prenatal ultrasound in 18^+4^th week of pregnancy showed absence of cerebellar vermis, dandy-walker malformation, hydrocephalus, dextrocardia, *situs inversus*, ventricular septal defect (0.3 cm), and double outlet of fetal right ventricle with pulmonary artery stenosis (Table 1). Pregnancy was terminated at the 23rd week and no obvious abnormality in appearance was observed (Figure 1A). The sixth pregnancy (case 2) showed recurrence of malformations by prenatal ultrasound at the 20th week of fetal development including occipital encephalocele (1.3 × 0.8 cm) (Figure 2A) and intra-uterine growth restriction (Table 1) (Figures 1B,C).

**TABLE 1:** Clinical phenotype and related genetic variants in eight fetuses with ciliopathies from five unrelated families.

Family	Case	Age	OE	PD	PK	Other	Gene	Variant	Inheritance	ACMG	Disease
1	1	18^+4^ w	−	−	−	Situs inversus, CHD, DWM	*CPLANE1*	c.7939delC (p.H2647IfsTer51)	Hom	P	JBS
						CVA					
	2	20 w	+	−	−	IUGR, banana-shaped cerebellum, lemon head	*CPLANE1*	c.7939delC (p.H2647IfsTer51)	Hom	P	JBS
2	3	23^+4^ w	−	−	+	Pericardial effusion, LV separation, cerebellar dysplasia, lemon head	*TMEM67*	c.1175C > G (p.P392R)	F	LP	MKS
								c.2439G > T (p.A813A, splice)	M	P	
	4	24 w	+	+	+	Balkes pouch	*TMEM67*	c.1175C > G (P392R)	F	LP	MKS
								c.2439G > T (p.A813A, splice)	M	P	
3	5	26^+4^ w	–	–	–	Abnormal nasal bone, increased renal cortical echogenicity	*NPHP4*	c.3730C > T (p.Q1244X)	Hom	P	NPH
						IUGR					
4	6	17 w	−	+	−	Dilation of the renal pelvis, narrow chest, short limbs, CPC	*DYNC2H1*	c.152T > G (p.L51R) c.988C > T (p.R330C)	M	LP	SRTD
									F	P	
	7	28 w	−	+	−	Narrow chest, short limbs	*DYNC2H1*	c.152T > G (p.L51R) c.988C > T (p.R330C)	M	LP	SRTD
									F	P	
5	8	17 w	−	+	−	Short limbs, low-set ears, CHD	*DYNC2H1*	c.4267C > T (p.R1423C) c.7858C > T (p.R2620X)	F	P	SRTD
									M	P	

OE, occipital encephalocele; PD, polydactyly; PK, polycystic kidneys; DWM, Dandy-Walker malformation; CVA, cerebellar vermis agenesis; CHD, congenital heart disease; IUGR, intra-uterine growth retardation; CPC, choroid plexus cyst; LV, left ventricle; F, father, M, mother; Hom, homozygous; ACMG, American College of Medical Genetics and Genomics; P, pathogenic; LP, likely pathogenic; JBS, Joubert syndrome; MKS, Meckel syndrome; SRTD, short-rib thoracic dysplasia; NPH, nephronophthisis.

WES revealed case 1 with a novel homozygous frame shift variant of c.7939delC (p.H2647IfsTer51) in *CPLANE1* (NM_023073.3). Sanger sequencing confirmed homozygosity in the proband and the heterozygosity in each parent (Table 1). Case 2 was also confirmed with same variant in case 1. The c.7939delC was not present in HGMD, gnomAD, or 1000G databases.

### Family 2

Case 3 and case 4 were affected siblings conceived of unrelated healthy parents with two early unexplained miscarriages. Prenatal ultrasound in 23^+4^rd week of case 3 showed separation of left lateral ventricles (0.9 cm), abnormality of the cerebellum, low amniotic fluid volume (amniotic fluid index 1.5 cm, maximum depth 1.0 cm), pericardial effusion (0.3 cm), and enlarged echogenic kidneys (left kidney 5.7 × 2.8 cm, right kidney 5.4 × 2.8 cm) with renal cysts (Table 1). Case 4 was found with congenital balkes pouch, cystic kidneys, polydactyly, and occipital encephalocele (Table 1). The pregnancy was terminated at the 24th week of gestation. Because prenatal detection and labor induction were performed in another hospital, we only obtained DNA of the two cases without ultrasound images and postnatal fetus examination.

WES was selected by Family 2 and the result showed that case 4 had compound heterozygous variants in *TMEM67* gene (NM_153704.6): a missense variant at c.1175C > G (p.P392R) and a synonymous variant at c.2439G > T (p.A813A), which were inherited from the parents, respectively (Table 1). The c.1175C > G, not reported previously, was predicted as damaging by PolyPhen2, SIFT, PROVEAN, and Mutation Taster. Total population frequency of this variant is 3.23185e-05. The c. 2439G > T variant is located at the last base of exon 23 and was classified as a disease-causing variant in HGMD. It might abolish the donor splice site following *in silico* analysis via SpliceAI. The predicted score of a donor site loss via SpliceAI was 0.64. After this, the DNA of case 3 was subjected to molecular analysis by Sanger sequencing. The result revealed the c.1175C > G and c.2439G > T variants in the *TMEM67* gene in case 3.

### Family 3

Case 5 was the first pregnancy of unrelated healthy parents. Prenatal diagnosis was performed in the 20th week of pregnancy due to a 12 Mb deletion in chromosome 1 as indicated by noninvasive prenatal testing (NIPT). The Affymetrix CytoScan 750K SNP array was used for chromosomal microarray analysis (CMA) and the result showed a region of homozygosity in 1p36.33-p36.13 (arr[hg19] 1p36.33p36.13 (888,658_18,337,268) x2 hmz) involving no established imprinted genes. In 26^+4^th week, missing nasal bone, delayed growth and development, and increased echogenicity in both renal cortexes were indicated by ultrasound (Figure 2B and Table 1). Prenatal trio-WES was chosen for further detection. A homozygous variant of c.3730C > T (p.Q1244X) (chr1:5,925,248) in *NPHP4* (NM_001184.3) (Table 1) was detected. This variant, occurring in the region of homozygosity, was inherited from the father. These results indicated that a segmental uniparental disomy at 1p36.33-p36.13 inherited from the father caused the autosomal recessive NPH.

### Family 4

Case 6 and case 7 were affected sibling fetuses from a non-consanguineous family with a healthy 6-year-old daughter. Ultrasound imaging of case 6 revealed abdominal circumference (10.3 cm), a femur length of 1.3 cm (<5th percentile), a humerus length of 1.0 cm (<5th percentile), and polydactyly. The pregnancy was subsequently terminated at the 18th week of gestation (Figure 1D). Fetal X-ray (Figure 2C) showed bilateral shortened curved femora and disproportionately shortened tibiae. Metaphyseal flaring of femora, tibiae, and fibulae were indicated. Ultrasound imaging of case 7 at the 28th week of gestation from this family exhibited similar sonographic features including small short femur (1.2 cm), narrow chest, light band separation in double kidney collection system (0.8 and 0.9 cm), peritoneal effusion (deepest 0.5 cm), and polydactyly. We only obtained DNA of case 7 but failed to perform postnatal fetus observation.

WES of case 6 revealed the compound heterozygous variants of *DYNC2H1* (NM_001377.3): c.152T > G (p.L51R) in exon 1 and c.988C > T (p.R330C) in exon 6. Sanger sequencing confirmed that the c.152T > G was present in the mother, and the c.988C > T in the father (Table 1). Case 7 was also confirmed with variants as case 6 through Sanger sequencing (Table 1). The c.988C > T has been reported as a pathogenic variant (El Hokayem et al., 2012; Schmidts et al., 2013). The c.152T > G was not present in HGMD, gnomAD, or 1000G databases, and was predicted as deleterious following *in silico* analysis. In addition, the c.152T > G is localized to the region of nucleotide-binding site of the *DYNC2H1* gene, which is highly conserved in mouse, rat, and dog orthologs of *DYNC2H1* gene.

### Family 5

Prenatal ultrasound of case 8 (the first fetus from non-relative parents) in the 12th week of pregnancy showed abnormal NT (4.4 mm). Recheck in the 17th week showed multiple malformations with large abdominal circumference (12.1 cm), ventricular septal defect (0.08 cm), left ventricular dysplasia, valve thickening, and transposition of the great arteries (Table 1). Besides, increased bowel and kidney echo, and bone dysplasia (femur = 1.3 cm, humerus = 0.9 cm, narrow chest) (Figure 2D) were detected. Examination of the aborted fetus showed low-set ears, polydactyly, a swollen abdomen, and short limbs (Figures 1E,F). Compound heterozygous variants of c.4267C > T (p.R1423C) and c.7858C > T (p.R2620X) in *DYNC2H1* (NM_001377.3) were identified. We confirmed a heterozygosity in each parent by Sanger sequencing (Table 1). The c.4267C > T was a pathogenic variant in short-rib polydactyly syndromes (SRPS) (Zhang et al., 2018). The c.7858C > T was a novel nonsense variant causing a truncated protein.

### Genotypes–Phenotypes Analysis

Since *CPLANE1* and *TMEM67* variants show significant clinical overlap, genotype-phenotype relationship analysis between them could help discriminate related disorders.

*CPLANE1* was identified as causative of OFD6 (orofaciodigital syndrome VI) and JBS. More than 60 pathogenic variants in the *CPLANE1* gene have been identified (Srour et al., 2012; Lopez et al., 2014; Vilboux et al., 2017; Bonnard et al., 2018) and 66% of cases carried biallelic truncating variants (Bonnard et al., 2018). Penetrance of common organ systems involving brain, skeleton, and kidney diseases in *CPLANE1* patients were compared (Table 2) (Lopez et al., 2014; Vilboux et al., 2017; Bonnard et al., 2018). MTS was nearly 100% detected in both JBS and OFD6. Polydactyly was with higher penetrance (100%) in OFD6 than JBS. The penetrance of occipital encephalocele in both JBS and OFD6 was estimated to be about 20%. However, cystic kidneys, short limbs, and congenital heart disease (CHD) were rarely observed.

**TABLE 2:** The comparison of clinical phenotypes between *CPLANE1* and *TMEM67*.

Phenotype	*CPLANE1*	*TMEM67*
MTS	13/14 (JBS)	1/20 (MKS)
	12/12 (OFD6)	10/13 (JBS)
OE	6/26 (OFD6)	10/20 (MKS)
	2/15 (JBS)	1/13 (JBS)
CHD	1/12 (OFD6)	/
PD	1/15 (JBS)	3/20 (MKS)
	12/12 (OFD6)	0/22 (JBS)
RD	1/14 (JBS)	1/20 (MKS)
	0/12 (OFD6)	0/22 (JBS)
KD	0/14 (JBS)	11/22 (JBS)
	0/12 (OFD6)	14/20 (MKS)
LD	6/15 (JBS)	21/22 (JBS)
		11/12 (MKS)
DD	14/14 (JBS)	2/20 (MKS)
	4/4 (OFD6)	11/13 (JBS)

MTS, molar tooth sign; OE, occipital encephalocele; CHD, congenital heart disease; PD, polydactyly; RD, retinal disease; KD, kidney disease; LD, liver disease; DD, developmental delay; JBS, Joubert syndrome; MKS, Meckel syndrome; OFD6, orofaciodigital syndrome VI.

**FIGURE 3:**
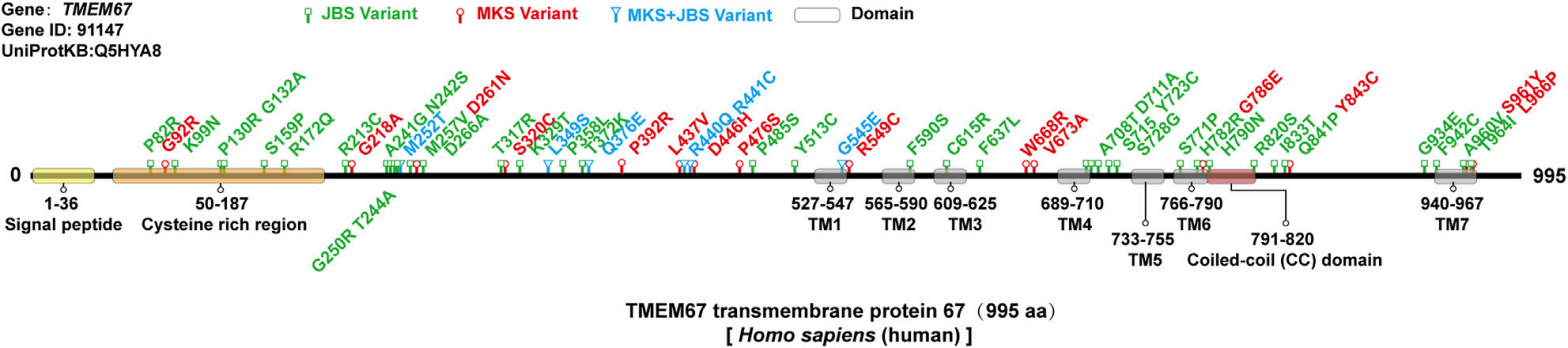
Missense variants of the *TMEM67* gene in MKS and JBS. There were about 58 (likely) pathogenic missense variants in the *TMEM67* gene identified thus far including 37 in JBS (green), 15 in MKS (red), and 6 in both (blue). The topology of *TMEM67*: a signal peptide (1–36 aa), a cysteine-rich region (50–187 aa), an extracellular area with unknown structure (188–526 aa), seven transmembrane regions (527–967 aa), and a short cytoplasmic tail (968–995 aa).

*TMEM67* was identified as causative of JBS and MKS. A genotype-phenotype correlation analysis based on a literature review shows that combination of two truncating variants in *TMEM67* gene is more common in lethal MKS than milder JBS (Iannicelli et al., 2010; Szymanska et al., 2012; Bachmann-Gagescu et al., 2015; Vilboux et al., 2017), which suggests that severity of variant changes disease outcomes. Thus, it is reasonable to presume that missense variant of *TMEM67* identified in MKS might bring more severe damage to protein function. There were about 58 (likely) pathogenic missense variants in *TMEM67* identified thus far including 37 in JBS, 15 in MKS, and 6 in both (Baala et al., 2007; Consugar et al., 2007; Khaddour et al., 2007; Brancati et al., 2009; Doherty et al., 2010; Iannicelli et al., 2010; Suzuki et al., 2016; Vilboux et al., 2017; Radhakrishnan et al., 2019) (Supplemental Table S1). Analysis of the topology of human *TMEM67* suggests the presence of a signal peptide (1–36 aa), a cysteine-rich region (50–187 aa), an extracellular area with unknown structure (188–526 aa), seven transmembrane regions (527–967 aa), and a short cytoplasmic tail (968–995 aa) (Figure 3) (Smith et al., 2006). Pathogenic missense variants of *TMEM67* mainly fall in the extracellular cysteine rich region, extracellular area with unknown structure, and the seven transmembrane regions (Figure 3). No pathogenic missense variant in the signal peptide and cytoplasmic tail has been reported (Figure 3). The comparison of their distribution along the gene in MKS versus JBS showed no obvious difference.

Two studies with larger *TMEM67* gene mutated cases were reviewed to analyze the characteristic of clinical phenotypes (Table 2) (Brancati et al., 2009; Szymanska et al., 2012; Vilboux et al., 2017). Incidence of liver and kidney diseases caused by *TMEM67* variants was high in both JBS and MKS. The prevalence of occipital encephalocele in MKS was higher than in JBS.

## Discussion and Conclusion

A clinical diagnosis of JBS can be made by brain imaging showing MTS. However, prenatal diagnosis of JBS has been proved to be difficult because of the relatively non-specific prenatal ultrasound findings. The clinical phenotypes of *CPLANE1* mutated cases in this report were highly heterogeneous, with intrafamilial variability. Meanwhile, case 1 and case 2 had no obvious MTS. Consistent with the absence of MTS in our fetuses, Doherty et al. described a JBS fetus with no obvious MTS phenotype instead of a deepening of the interpedullary fossa on MRI (Doherty et al., 2005)*.* Zhu et al. reported a prenatal JBS case with lemon sign and encephalocele (Zhu et al., 2021). *Situs inversus,* a specific feature in some MKS cases resulted from *MKS1* variants (Khaddour et al., 2007), was reported for the first time in our JBS case.

MKS is a perinatally lethal autosomal recessive condition characterized by central nervous system anomalies, hepatic defects, polycystic kidneys, and polydactyly (Radhakrishnan et al., 2019). Hepatic phenotype in the prenatal period could be detected at about the 15th to 26th week (Consugar et al., 2007). MKS fetuses in Family 2 were found with abnormal nervous system, polycystic kidneys, polydactyly but not hepatic defects at the 23rd and 24th weeks of pregnancy.

NPH is a major cause of pediatric end-stage renal disease and the phenotypes were mostly observed in children (Konig et al., 2017). It may be limited to the kidneys or can be associated with extrarenal organ. A study including 250 NPH patients showed that only 6/250 patients (2.4%) were detected with homozygous or compound heterozygous variants of the *NPHP4* gene (Hoefele et al., 2005). The prenatal diagnosis is one of the effective ways to avoid the birth of NPH children. However, prenatal NPH cases are rarely described. An NPH fetus with *NPHP1* variants manifested bilateral polycystic renal dysplasia and oligohydramnios at 16^+^th gestational week (Wu et al., 2020). As for the fetus in this study, antenatal ultrasonography showed missing nasal bone and increased echogenicity in both renal cortex in 26^+4^th week.

There are 111 pathogenic variants of the *DYNC2H1* gene that have been identified in 73 families (Zhang et al., 2018). Missense variants in the *DYNC2H1* gene had the highest frequency (71/111). Characterized phenotypes were polydactyly with a prevalence 30/73 (Zhang et al., 2018) and short limbs with a prevalence of 23/29 (Schmidts et al., 2013), while abnormal retina, kidney, and liver were rare (Schmidts et al., 2013). Prenatal cases in our report were distinguished by profound abnormalities of the skeleton, including markedly short ribs, extremely short limbs, and polydactyly.

In conclusion, this report expands prenatal clinical manifestations of ciliopathies and adds novel variants in *CPLANE1*, *TMEM67*, *NPHP4*, and *DYNC2H1* to the literature. Furthermore, detailed prenatal phenotypes of different ciliopathies provide evidence for prenatal WES testing.

## Data Availability

The data presented in the study are deposited in the China National GeneBank DataBase (CNGBdb) repository, accession number CNP0002142.
